# Psychosocial Well-Being of School-Aged Children Born to Bereaved (*Shidu*) Families: Associations with Mothers’ Quality of Life and Involvement Behaviors

**DOI:** 10.3390/ijerph17114166

**Published:** 2020-06-11

**Authors:** Ke Cui, Timothy Sim, Ting Xu

**Affiliations:** 1 School of Public Administration, Sichuan University, Chengdu 610065, China; xuting@scu.edu.cn; 2 Department of Applied Social Sciences, The Hong Kong Polytechnic University, Hung Hom, Kowloon, Hong Kong, China; timothy.sim@polyu.edu.hk

**Keywords:** psychosocial well-being, school-aged children, parental involvement, bereaved families, Wenchuan earthquake

## Abstract

Parents who lost their only child in the 12 May 2018 Wenchuan earthquake came to be known as the “*shidu*” (loss of an only child) parents. After the earthquake, they were beneficiaries of free reproductive health services, and most had another child. This study focuses on the psychosocial well-being of those children born to the *shidu* parents, and explores factors associated with mothers’ quality of life (QoL) and their involvement with their children. A cross-sectional survey was conducted in two primary schools in Wenchuan County. A sample of 192 families was analyzed (147 non-*shidu* and 45 *shidu*). The statistical analyses indicated that the children of *shidu* families had poorer peer relationships than children of non-*shidu* families. Moreover, *shidu* mothers’ expectations of their children’s achievements were significantly lower than for non-*shidu* mothers, but there was no statistically significant difference in the QoL between them. Additionally, a higher home-based involvement of mothers was found to be moderately associated with peer relationship problems of children in *shidu* families. Therefore, we suggest a future research focus on developing joint activities for parents and children that help to increase emotional communication for the psychosocial development of children in *shidu* families after disasters in China.

## 1. Introduction

On 12 May 2018, a devastating earthquake of 8.0 magnitude occurred in Wenchuan County, Sichuan Province, in southwest China. The Wenchuan earthquake was the most destructive earthquake in the history of the People’s Republic of China, causing more than 69,226 casualties, 374,643 injuries, and enormous economic losses [1,2]. Since the earthquake, a large body of research has been published on the mental disorders and psychological issues of women, as they have been identified as one of the most vulnerable groups for negative mental and physical health outcomes in disaster situations. However, the well-being of “*shidu*” mothers, women who lost their only child (“*shi*” means to lose and “*du*” means only child) in the disaster, have attracted little attention from social scientists. According to the provincial government, more than 8000 families lost their only child in the earthquake [3].

The number of one-child families has increased dramatically in China over the past 30 years due to the Chinese family planning one-child policy implemented in the late 1970s. In recent years, *shidu* has become a unique social phenomenon in Chinese society. Previous studies revealed that losing an only child means the termination of the major care-giving source for parents [4], their psychological and physical health may be impaired, and their social networks are weakened [5]. Losing an only child may even cause social isolation because parents may be stigmatized if they talk about their child’s death [6]. The one-child policy was eventually abandoned, and a two-child policy was implemented nationwide from 1 January 2016.

The concurrences of exposure to a disaster and the loss of a child were extremely stressful life events for women that could cause long-term health consequences. From 2010 to 2011, Xu et al. [7] conducted a mental health survey (i.e., anxiety, depression, PTSD, and complicated grief) with a group of women who had been affected by the Wenchuan earthquake and lost a child under the age of 18. They found that over 80% of these bereaved mothers experienced clinically significant psychological symptoms. Earlier research indicated that, compared to bereaved fathers, bereaved mothers were found to be more vulnerable to post-disaster functional impairment [8].

To support bereaved mothers and their families after the earthquake, the Standing Committee of Sichuan Provincial People’s Congress published “Decision on the reproduction of families with casualties in the Wenchuan earthquake” on 25 July 2008. Simply stated, the policy announced a series of free reproductive health services, including fertility assessments and infertility treatments, to assist *shidu* families in conceiving another child. By 2011, 3106 babies were delivered in Sichuan province as a result of the policy [9].

Quality of life (QoL) is widely used to refer to an individual’s perceptions of happiness, health status, and life satisfaction [10,11]. Exposure to natural disasters can impair a person’s QoL in the long-term [12,13,14]. Survivors who suffer from a disaster-induced mental impairment, such as PTSD, anxiety, and significant depression, also report a worse QoL than that of unaffected groups [15,16]. Furthermore, a cross-sectional survey among survivors of the Wenchuan earthquake revealed a worse QoL for women compared to men, and greater earthquake-related exposure was another risk factor for a decline in QoL [17]. However, the current literature on the Wenchuan earthquake rarely addresses the QoL status of *shidu* mothers. To the best of the author’s knowledge, thus far, only one study [18] in 2014 was conducted in the earthquake-stricken counties of Sichuan province to investigate *shidu* parents regarding their health-related QoL, but it did not examine the health disparities between mothers and fathers, nor did it survey the parents in relation to their happiness or life satisfaction.

Although previous research has paid significant attention to children who were directly exposed to the Wenchuan earthquake, few studies have investigated the psychosocial well-being of the children born to *shidu* parents after the Wenchuan earthquake. Various empirical evidence, based on research, has revealed the negative influence of maternal mental disorders, such as PTSD and depression, on the psychological adjustment and emotional and behavioral outcomes of children [19,20]. Specifically, a study conducted in 2012 with women who had been pregnant during the Wenchuan earthquake found that earthquake-related maternal psychiatric symptoms were negatively associated with their children’s mental development [21]. Furthermore, a recent study indicated that mothers’ psychological symptoms measured 12 and 18 months after the Wenchuan earthquake were longitudinally associated with their children’s poorer psychological status 10 years after the disaster [22]. Chen et al. [23] observed the developmental status of 164 *shidu* families 6 years after the Wenchuan earthquake, and reported that *shidu* parents, in general, perceived their children born after the earthquake to be “hyperactive” in comparison to their deceased children and to those from non-*shidu* families. In addition, Zhang [9] et al. explored the influencing mechanisms over children’s health status among the same cohort of *shidu* families, and they found that the mothers’ self-reported health condition and family income were significantly associated with their children’s general health status, while the impact of the fathers’ health condition was not statistically significant. However, no psychometric instruments were applied to assess the psychosocial problems of the children in these studies, nor did they systematically compare the psychosocial well-being of children born to *shidu* families with their peers living in a similar context.

Researchers have advocated using the concept of QoL to investigate its influence on behavioral problems in children. For example, a cross-sectional study involving low-income Chinese families in Hong Kong revealed that children (aged 6–12) whose mothers presented poorer health-related QoL had significantly higher psychosocial difficulties [24]. Parenting behavior has been shown to be a mechanism through which the poor QoL of parents affects the psychosocial well-being of children [25,26]. Specifically, in one study examining a group of primary school children in Hong Kong, Wong et al. [27] discovered that more parental involvement at home was associated with less overall behavioral problems in children, while higher levels of school-based parental involvement was associated with increased pro-social behaviors and reduced hyperactivity. In rural China, recent studies have documented that caregivers’ parenting knowledge and skills are significantly important for children’s social–emotional outcomes [28,29]. Moreover, women are shown to have primary childcare responsibility, especially in rural areas of China, as their husbands predominantly out-migrate for work [30]. However, the QoL status of *shidu* mothers in post-disaster contexts, their involvement in the education of their children, and their impact on the psychosocial well-being of children born after the disaster, are still unclear. The lack of relevant research regarding this unique group of Wenchuan earthquake survivors and their children has caused difficulties for social service practitioners, researchers, and policymakers when proposing programs and policies to support children’s life development in a sustainable fashion.

This study aimed to supplement knowledge regarding *shidu* families in China and contribute to ongoing research with culturally respectful, post-disaster psychosocial interventions. Essentially, the inquiry was guided by three hypotheses:

1. Children born to *shidu* families display poorer psychosocial well-being than their non-*shidu* peers.
2. The QoL of *shidu* mothers is worse than that of non-*shidu* mothers.
3. The QoL and involvement behaviors of mothers are positively associated with better psychosocial development outcomes for the children of *shidu* families.

## 2. Materials and Methods

### 2.1. Procedures and Participants

The primary data for the present study were derived from a cross-sectional survey conducted for the needs assessment of a social service project aimed at providing psychosocial interventions for *shidu* families after the Wenchuan earthquake. In cooperation with two primary schools in Wenchuan County, data collection was carried out from November to December 2018. After formal, informed consent agreement was obtained by the school administrators, parents and primary caregivers of all students in grades one, two, and three were invited into their schools to complete the survey (paper copy). Our intention was that at least one parent from each family would be able to attend the meeting, and these were held on a school day.

The purpose of the survey and the ethics protocols approved by an institutional review board were clearly explained in the survey introduction. In total, 306 (85.7%) of the 357 eligible participants attended the survey meetings. Of the 306 questionnaires returned, 295 (96.4%) were deemed valid, while the others were deemed invalid because they were substantially incomplete. Of the completed surveys, those submitted by mothers (*n* = 192) were selected for the purpose of this research. Fathers submitted 67 questionnaires, and the others (*n* = 36) were filled in by other caregivers. Therefore, 192 mother–child paired observations were ultimately included in the analysis.

### 2.2. Measures

The survey questionnaire used for this study included four sections. Section 1 requested socio-demographic information for the mother, father, and child (e.g., gender, age, ethnicity, maternal status, family income, education levels, etc.). The Strengths and Difficulties Questionnaire (SDQ) (parent version) was incorporated in order to assess the psychosocial well-being of children in Section 2. The SDQ is a 25-item behavioral screening scale developed to measure psychological adjustment and pro-social behavior in children and youths aged 4–17 years [31]. It consists of four negative behavior subscales, including conduct problems, hyperactivity, emotional symptoms, and peer relationship problems, and one subscale of pro-social behaviors, with each subscale comprising five items. Each item is rated on a three-point Likert scale (0 = Not true, 1 = Somewhat True, and 2 = Certainly true). The sum of the four negative behavior subscales is used to represent an overall difficulty score, with higher scores indicating greater difficulty. The pro-social scale indicates the degree of pro-social characteristics exhibited by a child. The parent version of the SDQ was previously applied and validated to the child population from eight provinces in mainland China (Cronbach’s alpha coefficient is 0.73 for the entire scale) [32].

Section 3 was designed to assess three aspects of parental involvement, including home-based and school-based involvement and parental expectation for achievement [33]. Items for home-based and school-based involvement were selected from the culturally adapted 29-item Parental Involvement Questionnaire for Primary School Students (parent version) developed at the National Key Lab of Cognitive Neuroscience and Learning of Beijing Normal University, China [34,35]. The home-based involvement measurement includes eight items (Cronbach’s alpha coefficient is 0.61) pertaining to the frequency of the parental monitoring of children’s behaviors outside of school (e.g., setting limits on unreasonable demands), parent–child communication (e.g., discussing academic performance, sharing interests with the child), and parent–child activity (e.g., visiting museums and attending recreational activities together). Similarly, four items (Cronbach’s alpha coefficient is 0.79), related to parent–school contact, were selected to measure the school-based involvement of parents, for example, “I talk with teachers about my child’s school performance”. These items were rated on a four-point Likert scale ranging from 1 = Never to 4 = Often. In addition, parental expectations for achievement were measured by parental responses to the single question, “How would you describe your expectations of your child’s achievement?” The three options were “3 = Very high, that is, ‘I hope my child can be outstanding in every aspect’”, “2 = Ordinary, that is, ‘I have some expectations of my child but will not demand them’”, and “1 = None, that is, ‘I rarely have any expectations of my child’”.

With reference to a previous study that measured QoL [36], Section 4 comprised three questions about the respondent’s self-perceived happiness (“Overall, do you currently feel happy with your life?”), general health status (“Overall, how do you feel about your health status?”), and life satisfaction (“Overall, do you currently feel satisfied with your life?”). Responses to the three questions were rated with a five-point Likert scale ranging from 1 to 5, representing increasing degrees for each indicator.

### 2.3. Analytical Strategies

All data analyses were implemented by IBM SPSS Statistics 22.0. Independent *t*-tests or chi-square tests were performed to compare the socio-demographic characteristics of mothers and children, psychosocial outcomes of children, maternal involvement, and QoL between the *shidu* and non-*shidu* families were observed. Unadjusted and adjusted linear regressions and ordinal logistic regressions were performed to further investigate the differences between *shidu* and non-*shidu* families regarding the studied variables. Missing data on mothers’ education (*n* = 3) were handled by mean substitution. In addition, Spearman’s correlation coefficients were generated to examine the unadjusted bivariate associations between the psychosocial outcomes of children and the QoL and involvement behaviors of mothers among *shidu* families (*n* = 45).

## 3. Results

### 3.1. Socio-Demographic Characteristics of the Research Participants

A total of 192 mother–child paired participants were observed in this study, of which mothers were the survey respondents. As shown in Table 1, 147 of the recruited families were non-*shidu* families, while 45 were *shidu* families. Although the sample size of *shidu* families was comparatively small, it comprised more than one fifth of the 203 children born to *shidu* families across Wenchuan County by the end of 2012 [37]. *Shidu* mothers were significantly older than non-*shidu* mothers (*p* < 0.000), about 11 years mean age difference. However, the two categories of mothers did not differ significantly in terms of education levels, marital status, or family income. As for the child participants, there were significant statistical differences in terms of gender (*p* = 0.005) and ethnicity (*p* = 0.001) between the two groups. It is worth noting that the percentages of boys (48.9%) and girls (51.1%) were approximately the same for *shidu* families. Moreover, no differences were found between the two groups of children in age and most of them were not the only child in the family.

### 3.2. Psychosocial Outcomes of Children, Maternal QoL, and Educational Involvement

Table 2 illustrates the psychosocial outcomes in five dimensions, as well as the total difficulty scores determined by the SDQ for all the children within the study. Table 2 also presents the comparative analysis results for the children of *shidu* families versus those from non-*shidu* families.

Hyperactivity (5.06 ± 2.18) appeared to be the most significant of all four dimensions of negative behaviors, while the dimension of pro-social behaviors obtained the highest average score (6.95 ± 1.78). Assessment results regarding pro-social behaviors for children of *shidu* and non-*shidu* families were nearly the same. Although the average score of total difficulty was slightly higher among children from *shidu* families, the difference was insignificant (*p* = 0.351). Regarding the four negative behavior subscales, which comprise the total difficulty score, no statistically significant differences in emotional problems, conduct problems, or hyperactivity were detected in the family groups. However, significant differences were identified between the two groups of children regarding peer relationship problems (*p* = 0.005). Specifically, children from *shidu* families reported more difficulty within peer relationships than children from non-*shidu* families.

Three indicators were used to measure the QoL status of mothers. As illustrated in Table 2, mothers of non-*shidu* families reported significantly better general health status than their counterparts. However, mothers from both groups did not differ significantly in terms of happiness and life satisfaction, though the reported life satisfaction among mothers from *shidu* families (4.49 ± 0.63) was slightly better. In addition, analyses revealed significant differences between mothers in both groups regarding school-based involvement (*p* = 0.024), and their expectations for their children’s achievements (*p* = 0.000). Specifically, mothers from *shidu* families reported significantly lower expectations and less frequent school-based involvement than mothers from non-*shidu* families. However, no significant difference was detected regarding the home-based involvement of mothers from both groups.

### 3.3. Unadjusted and Adjusted Regression Analyses

Several unadjusted and adjusted linear regressions and ordinal logistic regressions were performed to further explore the difference between family types (*shidu* families vs. non-*shidu* families). The key study variables that were detected as significantly different by family type using an independent *t*-test (illustrated in Table 2) were:

1. peer-relationship problems of children,
2. general health status of mothers,
3. school-based involvement,
4. expectations of achievement.

Table 3 and Table 4 display the results of the unadjusted and adjusted regression models.

The results indicated that children born to *shidu* families maintained significantly poorer peer relationships than their non-*shidu* counterparts, even when their gender, age, ethnicity, and only-child identity were controlled. Moreover, the general health status of mothers from *shidu* families was poorer with statistical significance in the unadjusted model, but the differences were insignificant when age, educational level, marital status, family income, and children’s characteristics were adjusted. Family income appeared to be positively associated with the general health status of mothers. Mothers’ expectations of children’s achievements consistently appeared to be significantly lower in *shidu* families, even when socio-demographic variables (i.e., mother’s age, educational level, marital status, family income, children’s gender, age, ethnicity, and only-child identity) were considered. However, the school-based involvement behavior of mothers was only associated with their marital status, as revealed from the adjusted model. 

### 3.4. Inter-Correlations of Study Variables among Shidu Families

Table 5 shows the inter-correlations of all study variables among *shidu* families (*n* = 45) using Spearman’s correlation coefficients.

The emotional problems of children were negatively correlated with maternal life satisfaction (r = −0.339, *p* < 0.05), while their pro-social behaviors were positively associated with maternal happiness (r = 0.458, *p* < 0.01). None of the other three negative psychosocial dimensions were revealed to be correlated with any of the three maternal QoL indicators with statistical significance. Nevertheless, the peer relationship problems of children were negatively related to maternal home-based involvement (r = −0.309, *p* < 0.05), which may indicate that the higher frequency of home-based involvement by mothers was associated with fewer peer-relationship problems for their children. However, the level of mothers’ home-based involvement did not appear to be correlated with any indicators measuring their QoL status. In contrast, maternal school-based involvement and expectations of achievement were positively correlated with their reported happiness (r = 0.333, *p* < 0.05) and general health status (r = 0.387, *p* < 0.01), respectively. It is worth noting there was a positive relationship between levels of home-based involvement and school-based involvement (r = 0.391, *p* < 0.01) by mothers, although neither of the two variables were associated with maternal expectations of child achievement. In addition, according to the effect size criteria outlined by Cohen [38], to evaluate the substantive significance of the correlations, all the above correlations were of medium strength.

## 4. Discussion

The primary purpose of this study was to examine the psychosocial well-being of children born to *shidu* families and compare them with children in the same age group from non-*shidu* families. Furthermore, the study compared mothers’ QoL and their involvement behaviors between *shidu* and non-*shidu* families, simultaneously exploring their associations with the psychosocial outcomes of children born to *shidu* families. The families included in this study were from two adjacent rural communities severely affected by the 2008 Wenchuan earthquake.

Our results only partially supported our research hypothesis that the psychosocial well-being of children from *shidu* families would be poorer than that of their non-*shidu* counterparts. Among the four negative behavior dimensions examined by the SDQ, statistical significance between the two groups of children was only found in regard to peer-relationship problems when demographic characteristics were considered. Peer-relationship problems in early childhood are worthy of attention, as they are not only associated with educational under-achievement and unemployment [39], but they may also lead to increased risk of externalizing behavior problems, such as criminality and substance abuse [40]. The persistence of disadvantages within peer relationships could predispose the children of *shidu* families to experience inferior developmental outcomes later in life.

The QoL status of mothers from *shidu* families was not found to be worse than that of the mothers from non-*shidu* families when socio-demographic information was controlled. Those bereaved mothers might have benefited from the timely policy that helped them to conceive another child. A new baby could bring great emotional comfort to a bereaved mother and help her to work through grief with her family and maintain bonds with the dead child [41]. Professional interventions, such as those provided by social workers for bereaved mothers immediately after the Wenchuan earthquake, might have contributed to their enhanced psychosocial well-being [42,43]. Although mothers of *shidu* families were significantly older than those of non-*shidu* families, age did not appear to be associated with the mothers’ general health status in this study. The average age of *shidu* mothers participating in the current study was consistent with the observational findings (mean age was 40.65) of Zhang et al. [9], who surveyed 164 *shidu* families across three Wenchuan earthquake-stricken counties in 2013. It was found that the mothers of *shidu* families had already been raising a single child for a number of years prior to the earthquake, and they then joined the study after giving birth with the help of the government fertility assistance.

As revealed by the present study, a higher level of home-based involvement by mothers was related to fewer peer-relationship problems among children in *shidu* families. This result is inconsistent with Wong et al. [27], who surveyed 507 Chinese third-grade school children in Hong Kong, and found that children’s peer problems were unrelated to parental home-based involvement, while the correlation between children’s conduct problems, hyperactivity, and parental home-based involvement was significant. A possible reason could be that the questionnaire items addressing home-based involvement in Wong et al.’s [27] study were predominantly measuring the frequency of parents’ academic-based involvement strategies, which may result in limited influence upon the children’s internalization of issues, such as peer-relationship problems. However, home-based involvement items used in the current study did not solely look at the frequency of maternal support of children’s schoolwork and study, but they also pertained to parental interest toward topics that interest children. The frequency of parent–child joint activities, such as visiting museums and participating in other recreational activities, were also included. This result supports previous suggestions that parents should employ non-academic-based involvement strategies and provide more emotional support to intervene with their children’s internalizing issues [44].

Most significantly, mothers in *shidu* families were more likely to report lower expectations of achievement compared to non-*shidu* mothers. Parental expectation of achievement essentially involves the values and attitudes of parents toward their children’s education or occupation, and these typically enhance the academic achievement and social skills of children [26]. The comparatively lower expectations of achievement by *shidu* mothers can be explained from three perspectives. First, the self-reported general health status of mothers was positively related to their expectations of achievement, as revealed in this study. This implies that the health-related individual QoL for mothers with experience of bereavement could be an essential factor in determining their attitudes toward the social achievements of their children. Second, the low expectations of mothers could be related to their underlying psychological issues, as disastrous experiences typically cause people to feel a lack of control of their future, and therefore, they lack motivation for setting goals [45]. Lastly, the impact of losing a child can change a person’s life values and meaning, resulting in a greater concern for the physical and mental health of children than for social achievement [23]. However, it is possible that the single question posed in the current study to survey the mothers’ expectations of achievement for their children was too general to detect the nuances between values and attitudes. Qualitative studies designed with techniques such as life history interviews might suggest further exploration of parental expectations among *shidu* parents and potential influencing factors. Furthermore, a multidimensional cultural adaptive approach is suggested to assess the health-related QoL of bereaved mothers, for example, SF-12 and WHOQOL-BREF, in order to obtain an integrative understanding of their health status.

In addition to the previously mentioned measurements of maternal health status and expectations of achievement, several other limitations of this study should be noted. First, as the study adopted a cross-sectional design, causal relationships between the psychosocial outcomes of children, maternal QoL, and involvement behaviors cannot be established. Additionally, this study was conducted more than ten years after the Wenchuan earthquake, making it difficult to ascertain the changes in the QoL of mothers or their attitudes toward children over time. Thirdly, although the current research was proposed as an exploratory analytical work, the small sample size of “*shidu*” families restricted the researchers to conduct adjusted regression analysis to further examine the associations between maternal home-based involvement and the peer-relationship problems of children, which may have limited the inference of the findings. Finally, the study did not include data from the fathers in the analysis; this was partially because only a few fathers participated in the survey, as many were out-migrant working. Future research should also cover a larger sample of this group, since China News [46] has reported that there were 3542 babies born to families who sustained casualties as a result of the Wenchuan earthquake by the end of 2015. Additionally, it may be helpful to interview parents who have chosen not to or were unable to have children again following a disaster or critical incident.

## 5. Conclusions

In conclusion, this study suggests closer attention should be paid to the peer relationship problems of children born to *shidu* families. It emphasizes the need for joint activities between parents and children with increased emotional communication, as such interventions may be particularly beneficial for those children to be able to acquire essential social skills. This study further declares that the health status and achievement expectations of bereaved mothers are important facets worthy of continued critical analysis. With further exploratory study, findings may motivate and inform researchers and practitioners to address the psychosocial problems of bereaved mothers and their children who are born after disasters occur.

## Figures and Tables

**Table 1:** Descriptive statistics for the demographics of children and their family characteristics.

		Non-*Shidu*, *n* (%)	*Shidu*, *n* (%)	*p*-Value
*n* = 192		147 (76.6)	45 (23.4)	t/F
**Demographics of children**				
Age of child (mean ± SD)		7.78 ± 1.01	8.00 ± 0.91	0.182
Gender	Female Male	42 (28.6) 105 (71.4)	23 (51.1) 22 (48.9)	0.005
Ethnicity	Han Zang Qiang Others	45 (30.6) 76 (51.7) 25 (17.0) 1(0.7)	24 (53.3) 9 (20.0) 11 (24.4) 1 (2.2)	0.001
Only child	Yes No	34 (23.1) 113 (76.9)	15 (33.3) 30 (66.7)	0.170
**Family characteristics**				
Marital status	Married Divorced Remarried	138 (93.9) 5 (3.4) 4 (2.7)	41 (91.1) 0 (0) 4 (8.9)	0.093
Family income	Low Middle low Middle Upper middle	30 (20.4) 41 (27.9) 74 (50.3) 2 (1.4)	11 (24.4) 12 (26.7) 21 (46.7) 1 (2.2)	0.818
Age of mothers (mean ± SD)		31.67 ± 4.47	42.59 ± 5.25	0.000
Education level of mothers	Primary or less Secondary Tertiary Missing	46 (31.3) 88 (59.9) 10 (6.8) 3 (2.0)	16 (35.6) 23 (51.1) 6 (13.3) 0	0.293

**Table 2:** Psychosocial outcomes of children, maternal quality of life (QoL), and involvement in education. (*n* = 192).

	All Families *n* = 192	Non-*Shidu* *n* = 147	*Shidu* *n* = 45	*p*-Value
Variables	Mean (SD)			
Psychosocial Outcomes of Children				
Strengths and Difficulties Questionnaire (SDQ): Emotional problems	3.06 (2.00)	3.10 (2.03)	2.91 (1.94)	0.577
SDQ: Conduct problems	2.33 (1.60)	2.23 (1.68)	2.67 (1.28)	0.110
SDQ: Hyperactivity	5.06 (2.18)	5.12 (2.21)	4.84 (2.10)	0.456
SDQ: Peer problems	3.42 (1.74)	3.22 (1.70)	4.04 (1.77)	0.005
SDQ: Total difficulties	13.86 (4.94)	13.68 (4.97)	14.47(4.84)	0.351
SDQ: Pro-social behaviors	6.95 (1.78)	7.00 (1.72)	6.80 (1.96)	0.511
**Maternal QoL**				
Happiness	4.12 (0.63)	4.12 (0.68)	4.11 (0.44)	0.573
Life satisfaction	4.34 (0.85)	4.29 (0.90)	4.49 (0.63)	0.174
General health status	3.69 (0.85)	3.77 (0.79)	3.42 (1.01)	0.039
**Maternal involvement in education**				
Home-based involvement	3.08 (0.41)	3.09 (0.42)	3.06 (0.38)	0.754
School-based involvement	2.66 (0.65)	2.72 (0.64)	2.47 (0.67)	0.024
Expectations of achievement	2.44 (0.54)	2.52 (0.52)	2.18 (0.54)	0.000

**Table 3:** Unadjusted and adjusted linear regression analyses for children’s peer problems and maternal school-based involvement (*n* = 192).

Variables	Peer Problems		School-Based Involvement	
	Model 1	Model 2	Model 3	Model 4
*Shidu* family	0.820 ** (0.292)	0.739 * (0.298)	−0.241 * (0.110)	−0.157 (0.157)
Boys		0.192 (0.281)		
Age of child		0.303 * (0.127)		
Ethnic minority		−0.361 (0.276)		
Only child		−0.257 (0.287)		
Age of mother				−0.007 (0.010)
Mother’s education				0.093 (0.082)
Married				−0.671 * (0.292)
Family income				−0.065 (0.058)
*F*	7.894 **	3.468 **	4.808 *	2.704 *
*R^2^*	0.040	0.085	0.025	0.068

Standard errors in parentheses; * *p* < 0.05, ** *p* < 0.01, *** *p* < 0.001.

**Table 4:** Unadjusted and adjusted ordinal logistic regression analyses for mothers’ general health status and expectations of achievement for their children (*n* = 192).

Variables	General Health Status		Expectations of Achievement	
	Model 5	Model 6	Model 7	Model 8
*Shidu* family	−0.704 * (0.320)	−0.750 (0.468)	−1.323 *** (0.377)	−1.243 * (0.531)
Boys		0.307 (0.317)		−0.057 (0.350)
Age of child		−0.074 (0.148)		−0.279 (0.163)
Ethnic minority		0.213 (0.309)		0.172 (0.340)
Only child		0.192 (0.322)		−0.153 (0.359)
Age of mother		0.017 (0.031)		−0.004 (0.034)
Mother’s education		−0.300 (0.253)		0.024 (0.279)
Married		−0.105 (0.873)		−0.358 (0.958)
Family income		0.579 ** (0.180)		−0.299 (0.195)
*χ2*	4.617 *	18.586 *	13.456 ***	19.712 *
*Pseudo R2*	0.026	0.101	0.086	0.124

Standard errors in parentheses; * *p* < 0.05, ** *p* < 0.01, *** *p* < 0.001.

**Table 5:** Inter-correlations of study variables among *shidu* families (*n* = 45).

	1	2	3	4	5	6	7	8	9	10	11
1. SDQ: Emotional problems	1.000	0.157	0.465 **	0.475 **	−0.226	−0.240	−0.339 *	−0.018	−0.239	−0.126	−0.014
2. SDQ: Conduct problems		1.000	0.017	0.387 **	−0.055	−0.148	0.079	0.246	−0.190	−0.182	−0.090
3. SDQ: Hyperactivity			1.000	0.151	0.042	−0.150	−0.119	−0.225	−0.065	−0.005	−0.237
4. SDQ: Peer problems				1.000	−0.081	−0.263	−0.163	−0.136	−0.309 *	−0.276	−0.018
5. SDQ: Pro-social behaviors					1.000	0.458 **	0.170	−0.136	0.176	0.208	−0.077
6. QoL: Happiness						1.000	0.438 **	0.163	0.072	0.333 *	0.316 *
7. QoL: Life satisfaction							1.000	0.327 *	0.160	0.029	0.094
8. QoL: General health status								1.000	0.128	0.149	0.387 **
9. Home-based involvement									1.000	0.391 **	0.034
10. School-based involvement										1.000	0.092
11. Expectations of achievement											1.000

* *p* < 0.05, ** *p* < 0.01, *** *p* < 0.001.
